# Bone Healing in Rabbit Calvaria Defects Using a Synthetic Bone Substitute: A Histological and Micro-CT Comparative Study

**DOI:** 10.3390/ma11102004

**Published:** 2018-10-17

**Authors:** Minas Leventis, Peter Fairbairn, Chas Mangham, Antonios Galanos, Orestis Vasiliadis, Danai Papavasileiou, Robert Horowitz

**Affiliations:** 1 Laboratory of Experimental Surgery and Surgical Research N. S. Christeas, Medical School, University of Athens, 75 M. Assias Street, Athens 115 27, Greece; orestis@vasiliadis.net (O.V.); d.pap.mes@gmail.com (D.P.); 2 Department of Periodontology and Implant Dentistry, School of Dentistry, University of Detroit Mercy, 2700 Martin Luther King Jr Boulevard, Detroit, MI 48208, USA; peterdent66@aol.com; 3 Manchester Molecular Pathology Innovation Centre, The University of Manchester, Nelson Street, Manchester M13 9NQ, UK; D.C.Mangham@manchester.ac.uk; 4 Laboratory of Research of the Musculoskeletal System, Medical School, University of Athens, 2 Nikis Street, Athens 145 61, Greece; galanostat@yahoo.gr; 5 Departments of Periodontics, Implant Dentistry, and Oral Surgery, New York University College of Dentistry, 345 E 24th Street, New York, NY 10010, USA; rah7@nyu.edu

**Keywords:** bone regeneration, β-tricalcium phosphate, calcium sulfate, bone substitutes, animal study

## Abstract

Bioactive alloplastic materials, like beta-tricalcium phosphate (β-TCP) and calcium sulfate (CS), have been extensively researched and are currently used in orthopedic and dental bone regenerative procedures. The purpose of this study was to compare the performance of EthOss versus a bovine xenograft and spontaneous healing. The grafting materials were implanted in standardized 8 mm circular bicortical bone defects in rabbit calvariae. A third similar defect in each animal was left empty for natural healing. Six male rabbits were used. After eight weeks of healing, the animals were euthanized and the bone tissue was analyzed using histology and micro-computed tomography (micro-CT). Defects treated with β-TCP/CS showed the greatest bone regeneration and graft resorption, although differences between groups were not statistically significant. At sites that healed spontaneously, the trabecular number was lower (*p* < 0.05) and trabecular separation was higher (*p* < 0.05), compared to sites treated with β-TCP/CS or xenograft. Trabecular thickness was higher at sites treated with the bovine xenograft (*p* < 0.05) compared to sites filled with β-TCP/CS or sites that healed spontaneously. In conclusion, the novel β-TCP/CS grafting material performed well as a bioactive and biomimetic alloplastic bone substitute when used in cranial defects in this animal model.

## 1. Introduction

Bone grafting procedures are performed to manage osseous defects of the jaw due to pathological processes or trauma, to preserve the alveolar ridge after extraction, and to augment the bone around dental implants. For this purpose, a wide variety of bone substitutes, barrier membranes, and growth-factor preparations are routinely used, and several different surgical methods have been proposed [1,2]. Autogenous bone is still considered the gold standard among bone grafting materials as it possess osteoconductive, osteoinductive, and osteogenetic properties; it neither transmits diseases nor triggers immunologic reactions; and is gradually absorbed and replaced by newly-formed high quality osseous tissue. The disadvantages of using autogenous bone include restricted availability, the need for additional surgical site, increased morbidity, and extended operating time [3,4]. As an alternative solution, bone graft substitutes are widely used in bone reconstructive surgeries, and the science of biomaterials has become one of the fastest growing scientific fields in recent years [5]. By definition, bone substitutes are any “synthetic, inorganic or biologically organic combination which can be inserted for the treatment of a bone defect instead of autogenous or allogenous bone” [6]. This definition applies to a wide variety of materials of different origins, composition, and biological mechanisms of function regarding graft resorption and new bone formation. Thus, the selection of biomaterials in clinical practice must be based on their biocompatibility, biodegradability, bioactivity, and mechanical properties, as well as the resulting cell behavior [7,8,9,10,11]. Parameters like the physicochemical characteristics, hydrophilicity and hydrophobicity, and molecular weight may influence the handling and performance of bone substitutes [12,13]. In general, the ideal grafting material should act as a substrate for bone ingrowth into the defect, to be ultimately fully replaced by host bone with an appropriate degradation rate in relation to new bone development for complete regeneration up to the condition of *restitutio ad integrum* [1,14]. The grafting material should also be able to retain the volume stability of the augmented area [1].

Bioactivity is a characteristic of chemical bonding between bone grafts and host biological tissues. Calcium phosphate ceramics and calcium sulfates are considered bioactive materials as they have the ability to evoke a controlled action and reaction to the host tissue environment with a controlled chemical dissolution and resorption, to ultimately be fully replaced by regenerated bone [5,15].

Among bioactive ceramics, β-TCP and hydroxyapatite (Ca10(PO4)6(OH)2) are frequently utilized in dental bone regenerative procedures [13]. Their composition is similar to that of natural bone, they are biocompatible and osteoconductive materials, can osseointegrate with the defect site, and due to their non-biologic origin, their use does not involve any risk of transmitting infections or diseases [16,17,18,19,20,21,22]. The degradation process of these biomaterials produces and releases ions that can create an alkaline environment that seems to enhance cell activity and accelerate bone reconstruction [13]. Recent in vitro and in vivo experimental studies have shown that such alloplastic bone substitutes can stimulate stem cells to differentiate to osteogenic differentiation of stem cells, as well as ectopic bone induction [23,24,25,26,27]. β-TCP may promote the proliferation and differentiation of endothelial cells and improve neovascularization in the grafted site, having clear benefits for osteogenic processes [13,28].

The ability of the bacteriostatic CS to set is well documented. Adding CS to β-TCP produces a compound alloplastic biomaterial that hardens in situ and binds directly to the host bone, helping maintain the space and shape of the grafted site, and acts as a stable scaffold [29,30,31,32,33,34,35]. The improved mechanical stability of the graft is a crucial factor for bone healing and differentiation of mesenchymal cells to osteoblasts [36], thus contributing to enhanced regeneration of high quality hard tissue [37,38]. The in situ hardening CS element may act as a cell occlusive barrier membrane, halting soft connective tissue proliferation into the graft during the first stages of healing [39,40,41].

Both CS and β-TCP are fully resorbable bone substitutes, leading to the regeneration of high quality vital host bone without the long-term presence of graft remnants. The CS element resorbs over a three- to six-week period, depending on patient physiology, creating a vascular porosity in the β-TCP scaffold for improved vascular ingrowth and angiogenesis. The β-TCP element resorbs by hydrolysis and enzymatic and phagocytic processes, usually over a period of 9–16 months. Although evaluating these resorptive mechanisms is difficult, it seems that cell-based degradation might be more important than dissolution, and macrophages and osteoclasts may be involved in phagocytosis, again largely dependent on host physiology [22,41,42,43].

As recent studies in bone reconstruction are gradually shifting their focus to biodegradable and bioactive materials, resorbable alloplastic bone substitutes might be a potential alternative to autogenous bone or bovine xenografts in dental bone reconstructive procedures. However, limited information is available in the recent literature. Therefore, the aim of this study was to compare the performance of a novel alloplastic bone substitute composed of β-TCP and CS, versus a bovine xenograft and spontaneous healing, in cranial bone defects in rabbits.

## 2. Experimental Section

### 2.1. Animals

Six adult male New Zealand White rabbits, each weighing 3 kg (±250 g), were used in this study with the approval of the Institutional Animal Care and Use Committee of the Veterinary Department, Greek Ministry of Rural Development and Veterinary, Attica Prefecture, Greece (project identification code: 5176/10-10-2017). Animals were provided with an appropriate balanced dry diet and water ad libitum, and caged individually in a standard manner at the N. S. Christeas animal research facility, Medical School, University of Athens, Greece. All animals were allowed seven days from their arrival to the facility in order to acclimatize to their new environment.

### 2.2. Surgical Procedures

**Figure 1:**
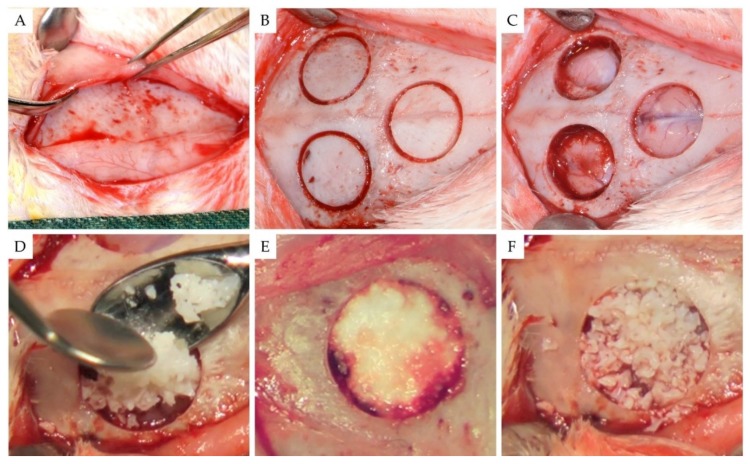
The surgical process. (**A**) Surgical exposure of the rabbit calvaria; (**B**) using a trephine burr, three identical circular osteotomies were performed; and (**C**) after removing the bicortical bone segments. The circular three-defect model was utilized with a frontal bone defect affecting the inter-frontal suture plus two bilateral defects affecting the parietal bones. (**D**) Two sites were treated with bone substitutes and the third left unfilled. (**E**) EthOss and (**F**) Bio-Oss.

Surgical procedures are shown in Figure 1. Under general anesthesia by orotracheal intubation, a longitudinal midline linear incision was made in the skin over the top of the cranial vault to expose the skull. The overlying periosteum was then excised, and three separate and identical 8-mm-diameter bicortical cranial round defects were created in the calvaria of each animal using a trephine drill with an internal diameter of 8 mm (Komet Inc., Lemgo, Germany) on a slow-speed electric handpiece by applying 0.9% physiologic saline irrigation. During the osteotomy, care was taken not to injure the dura mater under the bone. Then, using a thin periotome, the circular bicortical bone segment was mobilized and luxated.

Following a randomization technique using cards, the three resultant bone defects in each animal were randomly assigned treatment: (1) one defect was filled with 150 mg of the test alloplastic biomaterial (group 1), (2) one defect was filled with 150 mg of bovine xenograft (group 2), and (3) one sham defect remained unfilled (group 3).

The test bone graft substitute used in group 1 (EthOss, Ethoss Regeneration Ltd., Silsden, UK) is a self-hardening biomaterial consisting of β-TCP (65%) and CS (35%), preloaded in a sterile plastic syringe. In accordance with the manufacturer’s instructions, prior to applying the alloplastic graft into the bone defect, the particles of the biomaterial were mixed in the syringe with sterile saline. After application, a bone plunger was used to gently condense the moldable graft particles in order to occupy the entire volume of the site up to the level of the surrounding host bone. A saline-wet gauze was used to further compact the graft particles and accelerate the in situ hardening of the CS element of the graft. As a result, after a few minutes, the alloplastic bone substitute formed a stable, porous scaffold for host osseous regeneration.

As a xenograft, a bovine deproteinized cancellous bone graft with a particle size of 0.25–1 mm (Bio-Oss, Geistleich Pharmaceutical, Wollhausen, Switzerland) was used in group 2. Bio-Oss consists of loose particles. According to the manufacturer’s instructions, before application, the material was mixed with sterile saline and then placed into the bone defect, avoiding excessive compression.

Interrupted resorbable 4-0 sutures (Vicryl, Ethicon, Johnson & Johnson, Somerville, NJ, USA) were used to close the overlying soft tissues in layers.

Each experimental animal received antibiotics (30 mg/kg of Zinadol, GlaxoWellcome, Athens, Greece) every 24 h and analgesics (15 mg/kg of Depon; Bristol-Myers Squibb, Athens, Greece) for 2 days postoperatively. An intravenous injection of sodium thiopental (100 mg/kg of Pentothal; Abbott Hellas, Athens, Greece) was used to euthanize all animals after an 8-week healing period. The calvaria bones containing the healed sites were surgically harvested and immediately fixed in neutral buffered formalin (10%) for 24 h.

### 2.3. Micro-CT Evaluation

Each calvaria was scanned using a micro-CT scanner (Skyscan 1076, Bruker, Belgium) at 50 kV, 200 μA, and a 0.5 mm aluminum filter. The pixel size was 18.26 μm. Two images were captured every 0.7° through 180° rotation of the sample; the exposure time per image was 420 ms. The X-ray images were reconstructed using the NRecon software (Skyscan, Bruker, Belgium) and analyzed using Skyscan CT analysis software. Specific thresholds were set on segmenting the micro-CT images in order to distinguish the newly-formed bone from the connective tissue and the grafting materials. A lower threshold (level 60) was used for all groups to segment the bone tissue, whereas higher threshold levels were used to segment the Bio-Oss and the EthOss particles (level 90 and level 120, respectively). Analysis was performed using an 8-mm-diameter circular region that was placed in the center of the initial defect area. Trabecular bone analysis was performed, and based on the micro-CT results several parameters regarding new bone formation, residual graft, and the microarchitecture of the regenerated bone were calculated (Table 1).

### 2.4. Histology

After micro-CT analysis, bone specimens were decalcified in bone decalcification solution (Diapath S.p.a., Martinengo, Italy) for 14 days. After routine processing, slices were obtained from the central part of each healed bone defect using a saw (Exakt saw 312, Exakt Apparatebau GmbH, Norderstedt, Germany), embedded in paraffin, sectioned longitudinally into multiple 3-μm-thick sections and stained with Hematoxylin and Eosin stain. For qualitative analysis of the bone regenerative process, the stained preparations were examined under a light microscope (Nikon Eclipse 80, Nikon, Tokyo, Japan) and the entire section was evaluated. Images of each section were acquired with a digital camera microscope unit (Nikon DS-2MW, Nikon, Tokyo, Japan).

### 2.5. Statistics

Statistical analysis was performed using SPSS software (v. 17, SPSS Inc., Chicago, IL, USA). Data are expressed as mean ± standard deviation (SD). The Shapiro-Wilk test was utilized for normality analysis of the parameters. The comparison of variables among the 3 groups was performed using the one-way ANOVA model. Pairwise comparisons were performed using the Bonferroni test. All tests were two-sided, and statistical significance was set at *p* < 0.05.

## 3. Results

### 3.1. Overall

**Figure 2:**
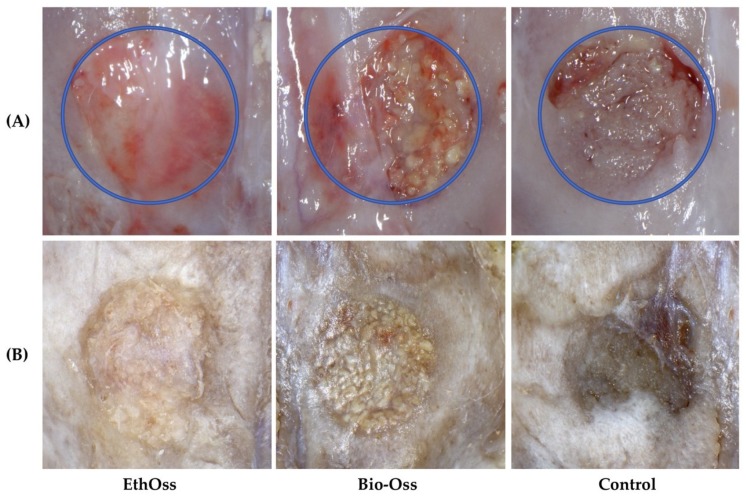
Gross observations of the 8-mm-diameter calvaria bone defect sites after eight weeks of healing: (**A**) fresh harvested rabbit calvaria and (**B**) after removing the dura mater and fixed in neutral buffered formalin (10%) for 24 h. Clinical observation revealed a different pattern of healing of the osseous defect between groups.

All animals survived for the duration of the study without complications. At eight weeks, there were no clinical signs of infection, hematoma, or necrosis at the defect sites. The dura mater and brain tissues under all bone defect sites exhibited no clinical evidence of inflammation, scar formation, or an adverse tissue reaction to the bone grafting materials (Figure 2A). Closure of the cortical window and filling of the defects with new bone were macroscopically observed at all defect sites. At bone defect sites grafted with bovine xenograft (Bio-Oss), graft particles embedded in newly formed hard tissue were clinically observed, whereas the newly-formed hard tissue occupying the sites treated with the alloplastic biomaterial (EthOss) was macroscopically homogeneous, without clear clinical distinction of residual graft particles. Clinical observation of sites left empty revealed that the spontaneously healed circular bone defects were bridged by a thin layer of newly-formed hard tissue (Figure 2B, Video S1).

### 3.2. Micro-CT Evaluation

**Figure 3:**
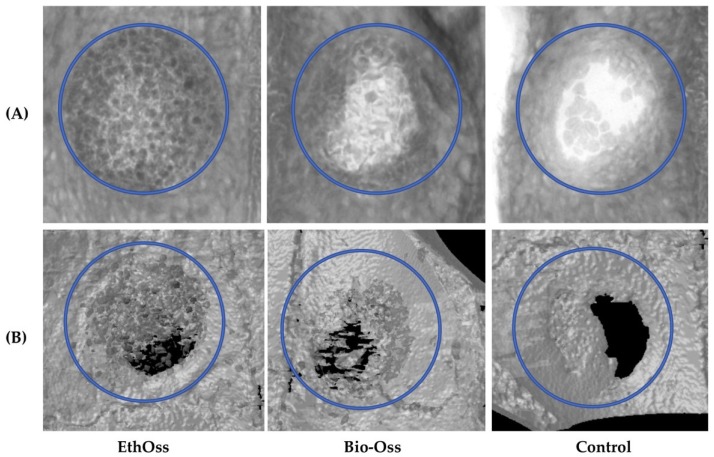
(**A**) Axial sections and (**B**) reconstructed three-dimensional (3D) micro-computed tomography (CT) images of the 8-mm-diameter defect sites after eight weeks of healing.

The radiological imaging results acquired from the micro-CT after eight weeks of healing are shown in Figure 3.

**Figure 4:**
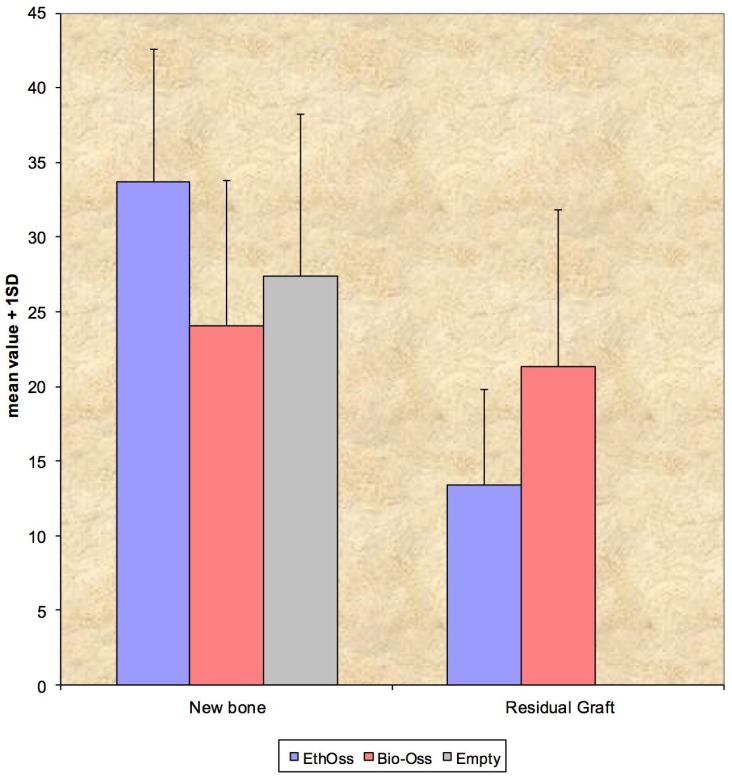
The percentage of new bone (BV/TV) between the three groups, and the percentage of residual graft (RMVF) in sites treated with EthOss and Bio-Oss, after eight weeks of healing. Data are presented as means. The differences between groups were not statistically significant (*p* > 0.05).

There were no significant differences between parameters that expressed new bone regeneration (BV/TV), between the three groups. At this time point, there were no statistically significant differences regarding the percentage of the residual graft material (RMVF) between groups 1 and 2, where EthOss and Bio-Oss were used as bone substitutes, respectively (Figure 4 and Table 2).

At eight weeks, there were statistically significant differences between the three groups in the parameters associated with the microarchitecture of the newly-formed hard tissue (Table 3). Regarding the parameter Tb.N, pairwise comparisons indicated statistically significant difference between group 3 (Empty) and group 1 (EthOss) (*p* < 0.001), and group 2 (Bio-Oss) (*p* < 0.001), whereas there was no difference between group 1 (EthOss) and group 2 (Bio-Oss) (*p* = 0.126). Regarding the parameter Tb.Th, pairwise comparisons indicated statistically significant difference between group 2 (Bio-Oss) and group1 (EthOss) (*p* = 0.001), and Empty (*p* < 0.001), whereas there was no difference between group 1 (EthOss) and group 3 (Empty) (*p* = 1.000). For parameter Tb.S, pairwise comparisons indicated statistically significant difference between group 3 (Empty) and group 1 (EthOss) (*p* = 0.001), and group 2 (Bio-Oss) (*p* < 0.001), whereas there was no difference between group 1 (EthOss) and group 2 (Bio-Oss) (*p* = 0.662).

### 3.3. Histology

**Figure 5:**
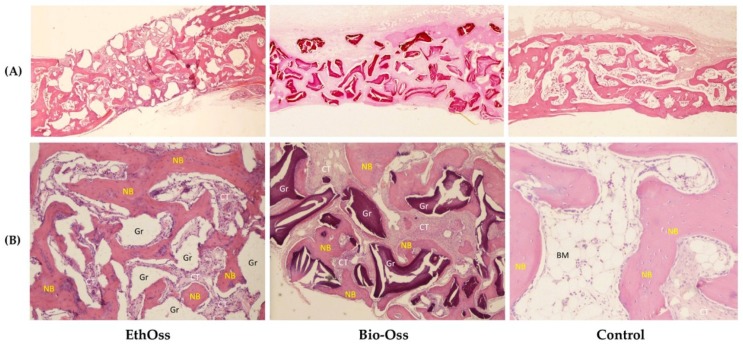
Histological specimens at eight weeks of healing (Hematoxylin and Eosin staining). (**A**) Cross-sections of the grafted and nongrafted sites (original magnification 5×); (**B**) EthOss and Bio-Oss particles (Gr) are embedded in well-perfused connective tissue (CT) and newly-formed bone (NB). Control group showing newly-formed bone trabeculae, bone marrow, and connective tissue (original magnification 50×).

The histological slides for groups 1 (EthOss) and 2 (Bio-Oss) after eight weeks of healing are shown in Figure 5. Histologically, the analyzed biopsy contained newly-formed bone, residual grafting material, and vascularized uninflamed connective tissue. In all specimens, no significant inflammatory response, no necrosis, or foreign body reactions were observed. The graft particles were surrounded by or in contact with lamellar bone, demonstrating good osteoconductivity and biocompatibility.

## 4. Discussion

The aim of this animal study was to evaluate the host response after implantation of a novel bioactive and fully resorbable alloplastic grafting material in comparison to the effect of a bovine xenograft or spontaneous natural healing, in surgically-created calvaria bone defects in rabbits.

The β-TCP/CS material was compared to a bovine xenograft. Anorganic bovine bone substitutes have been extensively studied and used in oral surgery and implantology. Numerous pre-clinical studies and clinical trials in dentistry have shown and described in detail their osteoconductive properties and their ability to maintain the volume of the augmented site in the long-term [44,45,46,47,48,49,50]. However, controversy still remains as to whether this graft source is truly resorbable [51,52]. Mordenfeld et al. [51] performed histological and histomorphometrical analyses of human biopsies that were harvested 11 years after sinus floor augmentation with deproteinized bovine and autogenous bone. They found that the xenograft particles were not resorbed but were well-integrated in lamellar bone with no significant changes in particle size. Another important issue is that there are still significant concerns that bone grafts of bovine origin may carry a possible risk of transmitting prions to patients [53]. According to Kim et al. [54], screening prions within the animal genome is difficult. Moreover, there is a long latency period to manifestation of bovine spongiform encephalopathy (from one year to over 50 years) in infected patients. These factors provide a framework for the discussion of possible long-term risks of the xenografts that are used so extensively in dentistry. Thus, the authors suggested abolishing the use of bovine bone. They also highlighted that patient counseling should always include a clear description of the bone grafting material origin in bone reconstructive procedures.

In our study, no fibrosis developed between the particles of the biomaterial and the regenerated bone, nor was an inflammatory response observed, confirming the biocompatibility of EthOss. Our results indicate that β-TCP/CS can support new bone formation in parallel with biomaterial dissolution. The test alloplastic graft (β-TCP/CS = 65/35) presented in this study showed pronounced new bone formation (BV/TV = 33.70%) at eight weeks after implantation in circular calvaria bone defects in rabbits. Previous experimental animal studies using similar β-TCP/CS materials reported new bone fractions varying from 26% to 49% after a healing period of three weeks to four months [32,33]. Yang et al. [55], using micro-CT analysis to study the performance of a β-TCP/CS bone substitute in a sheep vertebral bone defect model, reported a 59% hard tissue volume at 36 weeks.

The degradation of β-TCP/CS biomaterials has been demonstrated in other pre-clinical studies. Using histomorphometry, Leventis et al. [33] found a statistically significant decrease in the percentage of residual material between three and six weeks (4.54% and 1.67%, respectively) in grafted rabbit calvaria defects. Podaropoulos et al. [32] reported 21.62% of residual β-TCP/CS four months after implantation of the material in surgically created bone defects on the iliac crest of Beagle dogs, whereas complete biodegradation of the β-TCP/CS graft after 36 weeks of implantation was documented in a previous animal study [55]. In accordance with the above findings, the present study demonstrated the degradation of the β-TCP/CS test biomaterial, showing a mean graft fraction area of 13.41% at eight weeks post-implantation.

In a clinical report, Fairbairn et al. [35] used β-TCP/CS for alveolar ridge preservation. Twelve weeks after socket grafting, a trephine biopsy was performed before the placement of the implant, and the authors histologically and histomorphometrically analyzed the sample of the regenerated bone, revealing 50.28% newly-formed bone and 12.27% remnant biomaterial.

In this study, we used micro-CT to three-dimensionally observe the structure of the newly-formed bone and to analyze important parameters of bone architecture. Micro-CT is a non-invasive, non-destructive analytical method that allows a significantly larger region of interest in the sample to be directly analyzed in three dimensions, compared to traditional histological methods. In combination with the histological findings, a comprehensive image of the regenerated bone can be surveyed to provide a representative and complete description of the healing outcome in the defect site [56].

The analysis of the micro-CT data in the present study revealed statistically significant differences regarding parameters associated with the microarchitecture of the healed sites at eight weeks post-operation. At sites that healed spontaneously, the trabecular number was lower and trabecular separation was higher. These findings in the control group indicate that the grafting materials used for filling the bone defects in the other groups acted as an osteoconductive scaffold, which facilitated the development of a larger number of trabeculae in a denser three-dimensional arrangement. In parallel, defects treated with β-TCP/CS showed the greatest bone regeneration and graft resorption, although differences between groups were not statistically significant.

The use of grafting materials to treat bone defects might have an important effect on the amount of regenerated bone tissue, and the presence of the graft particles may alter the microarchitecture of the newly-formed tissue. The resorption rate and the ability of a given grafting material to assist bone reconstruction seem to affect the bone healing mechanism and the geometry of the newly-formed tissue. Such differences might affect the overall quality of the newly-formed bone [7]. The capacity of the regenerated bone to remodel and adapt to the transmitted occlusal forces, thus minimizing the risk of failure under load, depends on the amount of bone, as well as its shape and microarchitecture (spatial distribution of the bone mass) and the intrinsic properties of the materials that comprise this hard tissue. Although a moderate to strong correlation between trabecular bone volume/architecture and biomechanical properties of trabecular bone has been shown [57], it is still unclear how the long-term presence of remnant non-resorbable or slowly resorbable particles of the graft, and the associated differences in structural parameters of trabecular bone and bone microarchitecture, might interfere with the remodeling and the strength of the new tissue when regenerating bone around dental implants. In a systematic review of the alterations in bone quality after alveolar ridge preservation with different bone graft substitutes, Chan et al. [7] reported significant variations in vital bone formation utilizing different grafting materials and discussed the concern that the presence of residual biomaterial might interfere with normal bone healing and remodeling, reducing the bone-to-implant contacts, and possibly negative affecting the overall quality and architecture of the bone that surrounds the implants. However, whether changes in bone quality influence implant success and peri-implant tissue stability remains unknown.

The outcomes of the present study revealed the highest vital bone content for defects grafted with the test alloplastic material (33.70%), followed by sockets with no graft material (27.36%), and the bovine xenograft (24.07%), whereas the amount of residual graft was higher (21.36%) for the bovine xenograft compared to the alloplast (13.41%). Our findings, although not statistically significant, are in accordance with results from human clinical studies on flapless socket grafting. In a recent systematic review, Jambhekar et al. [10] analyzed the outcomes of randomized controlled trials that reported that, after a minimum healing period of 12 weeks, sockets filled with alloplastic biomaterials had the highest amount of newly-formed bone (45.53%) compared to sites subjected to spontaneous healing with no graft material (41.07%) and xenografts (35.72%). The amount of remnant biomaterial was highest for sites treated with xenografts (19.3%) compared to alloplastic materials (13.67%).

## 5. Conclusions

The present histological and micro-CT investigation of rabbit cranial bone defects treated with the test resorbable alloplastic β-TCP/CS graft demonstrated excellent biocompatibility of the biomaterial and pronounced new bone formation after a healing period of eight weeks.

## Figures and Tables

**Table 1:** Parameters assessed by analysis of the micro-computed tomography (CT) data.

Parameter	Abbreviation	Description	Standard Unit
Bone volume fraction	BV/TV	Ratio of the segmented newly-formed bone volume to the total volume of the region of interest	%
Residual material volume fraction	RMVF	Ratio of the residual grafting material volume to the total volume of the region of interest	%
Trabecular number	Tb.N	Measure of the average number of trabeculae per unit length	1/mm
Trabecular thickness	Tb.Th	Mean thickness of trabeculae, assessed using direct 3D methods	mm
Trabecular separation	Tb.Sp	Mean distance between trabeculae, assessed using direct 3D methods	mm

**Table 2:** Comparison of parameters associate with the newly-formed bone (BV/TV) and the residual grafting material (RMVF). The differences between groups were not statistically significant (*p* > 0.05).

Parameter	Site	N	Mean	SD	*p*-Value
BV/TV	EthOss	6	33.70	8.94	0.525
	Bio-Oss	6	24.07	9.69	
	Control	6	27.36	10.95	
RMVF	EthOss	6	13.41	6.43	0.070
	Bio-Oss	6	21.36	10.05	
	Control	6	-	-	-

**Table 3:** Comparison of parameters associated with the microarchitecture of the newly-formed hard tissue in the three groups (a: *p* < 0.05 vs. control; b: *p* < 0.05 vs. Bio-Oss).

Parameter	Site	N	Mean	SD	*p*-Value
Tb.N	EthOss	6	1.511 ^a^	0.255 ^a^	<0.001
	Bio-Oss	6	1.213 ^a^	0.198 ^a^	
	Control	6	0.541	0.239	
Tb.Th	EthOss	6	0.219 ^b^	0.018 ^b^	<0.001
	Bio-Oss	6	0.291	0.029	
	Control	6	0.210 ^b^	0.028 ^b^	
Tb.S	EthOss	6	0.486 ^a^	0.136 ^a^	<0.001
	Bio-Oss	6	0.713 ^a^	0.238 ^a^	
	Control	6	1.686	0.455	

## Supplementary Materials

The following are available online at http://www.mdpi.com/1996-1944/11/10/2004/s1, Video S1: Video of representative harvested samples fixed in neutral buffered formalin revealing macroscopically the different pattern of bone healing between groups.
